# Follow-up colonoscopy after an abnormal stool-based colorectal cancer screening result: analysis of steps in the colonoscopy completion process

**DOI:** 10.1186/s12876-021-01923-1

**Published:** 2021-09-28

**Authors:** Gloria D. Coronado, Alexandra Kihn-Stang, Matthew T. Slaughter, Amanda F. Petrik, Jamie H. Thompson, Jennifer S. Rivelli, Ricardo Jimenez, Jeffrey Gibbs, Neha Yadav, Rajasekhara R. Mummadi

**Affiliations:** 1 Center for Health Research, Kaiser Permanente Northwest, 3800 North Interstate Avenue, Portland, OR 97227 USA; 2 Oregon Health Sciences University, Portland, OR USA; 3 Sea Mar Community Health Centers, Seattle, WA USA; 4 Northwest Permanente Medical Group, Portland, OR USA

**Keywords:** Follow-up colonoscopy, Time to colonoscopy, Reasons for non-adherence, Colonoscopy pathway, Federally qualified health centers

## Abstract

**Background:**

Delays in receiving follow-up colonoscopy after an abnormal fecal immunochemical test (FIT) result are associated with increased colorectal cancer incidence and mortality. Little is known about patterns of follow-up colonoscopy completion in federally qualified health centers.

**Methods:**

We abstracted the medical records of health center patients, aged 50–75 years, who had an abnormal FIT result between August 5, 2017 and August 4, 2018 (N = 711). We assessed one-year rates of colonoscopy referral, pre-procedure visit completion, colonoscopy completion, and time to colonoscopy; associations between these outcomes and patient characteristics; and reasons for non-completion found in the medical record.

**Results:**

Of the 711 patients with an abnormal FIT result, 90% were referred to colonoscopy, but only 52% completed a pre-procedure visit, and 43% completed a colonoscopy within 1 year. Median time to colonoscopy was 83 days (interquartile range: 52–131 days). Pre-procedure visit and colonoscopy completion rates were relatively low in patients aged 65–75 (vs. 50–64), who were uninsured (vs. insured) or had no clinic visit in the prior year (vs. ≥ 1 clinic visit). Common reasons listed for non-completion were that the patient declined, or the provider could not reach the patient.

**Discussion:**

Efforts to improve follow-up colonoscopy rates in health centers might focus on supporting the care transition from primary to specialty gastroenterology care and emphasize care for older uninsured patients and those having no recent clinic visits. Our findings can inform efforts to improve follow-up colonoscopy uptake, reduce time to colonoscopy receipt, and save lives from colorectal cancer.

*Trial registration*: National Clinical Trial (NCT) Identifier: NCT03925883.

## Background

Colorectal cancer (CRC) is the third leading cause of cancer death in the United States, accounting for an estimated 51,000 deaths in 2019 [1]. Due to increased screening, CRC incidence and mortality has declined considerably over the past four decades, however many people who should be screened still do not get screened [2]. Fecal immunochemical tests (FITs) are a low-cost alternative to colonoscopy for identifying patients at risk of CRC, however, for FITs to be effective, patients who receive an abnormal FIT result must obtain a timely follow-up colonoscopy in order to remove precancerous polyps or find cancer in early forms that can be successfully treated. Troublingly, follow-up colonoscopy rates are low in most US healthcare settings and are particularly low in federally qualified health centers (FQHCs), where published rates range from 18 to 57% [3–7]. These rates are well below the 80% target set by the Multi-Society Task Force on Colorectal Cancer [8]. Compared with health care settings that have integrated specialty care services, FQHCs face unique challenges in coordinating care with specialty colonoscopy providers located in separate health care systems.

Delays in receiving follow-up colonoscopy are associated with increased CRC incidence and mortality. Lee and colleagues reported a 31% higher risk of CRC and a two-fold higher risk for advanced stage disease among adults who delayed colonoscopy by 6 months or more compared to those who obtained a colonoscopy within 1–3 months of an abnormal FIT [9]. Similar findings were reported in a Kaiser Permanente study, where a 10-month delay was associated with a 48% increase in CRC risk [10]. Meester and colleagues used modeling to estimate a 4% increase in CRC incidence and 16% increase in mortality among adults who delayed follow-up colonoscopy by 12 months versus those who received it within 2 weeks [11].

For many patients, obtaining a follow-up colonoscopy can be a complex process, often requiring the completion of several steps, including obtaining a referral to a gastroenterology (GI) practice; possibly attending a pre-procedure visit; arranging an escort and transportation to and from the procedure; completing bowel preparation; and attending the colonoscopy procedure. Each of these steps can create compounding barriers to colonoscopy completion. Analyses of qualitative interviews with patients have identified several barriers, including lack of knowledge about the need for colonoscopy, difficulty arranging transportation to and from the procedure, concerns about cost, inability to leave work to attend the procedure, fear of discomfort, and difficulty completing bowel preparation [12–14].

In a previous retrospective cohort study conducted in a San Francisco-based integrated safety net health system, Issaka and colleagues reported that among patients with an abnormal FIT result, 87% obtained a colonoscopy referral, 65% attended a pre-procedure visit, and 56% obtained a colonoscopy [6]. Notably, patients in this study were referred for colonoscopy to a single integrated hospital system, and little is known about patterns of participation across these colonoscopy completion steps in non-integrated health centers.

As part of the Predicting and Addressing Colonoscopy Non-adherence in Community Settings (PRECISE) study, we used data abstracted from the medical records of patients with an abnormal FIT result to explore patterns of follow-up colonoscopy completion and identify key steps in the process at which patients stopped seeking care (referral, pre-procedure visit, or colonoscopy completion) and reasons for this discontinuation. Our analysis builds on prior research by assessing patient characteristics associated with successful completion of intermediate steps, and reasons listed in the electronic health record (EHR) for discontinuation of each step. Moreover, our study was set in a non-integrated health center and our analysis includes more abstracted health records than prior research [6].

## Methods

The PRECISE study is an individual randomized controlled trial of patient navigation versus usual care for follow-up colonoscopy completion among patients who receive an abnormal FIT result at Sea Mar Community Health Centers [15]. As part of PRECISE, we conducted a baseline assessment of follow-up colonoscopy in a retrospective cohort of patients who had an abnormal FIT result over a one-year interval from August 5, 2017, to August 4, 2018. We abstracted the health records of these patients to assess completion of intermediate steps in obtaining a follow-up colonoscopy (i.e., referral to GI, completion of pre-procedure visit, and completion of colonoscopy procedure) and time to colonoscopy in the 365 days following the abnormal FIT result. The PRECISE study protocol, including this assessment, was reviewed, and approved by the Institutional Review Board at Kaiser Permanente Northwest [protocol # STUDY00000779]. We obtained a waiver of informed consent, given the minimal risks posed to patients. Our study adheres to CONSORT reporting requirements for clinical studies.

### Setting and study population

Sea Mar Community Health Centers is a large FQHC that operates 32 primary care clinics in western Washington State. Sea Mar provides a broad range of health and human services to a large and diverse population of more than 305,000 individuals across 13 counties, 37% of whom are Latinx. Annually, about 29,000 Sea Mar patients are age-eligible for FIT screening. In 2018, the Sea Mar CRC screening rate was 45%, and the average FIT positivity rate was 9%; thus, about 700 patients received an abnormal FIT test result. Sea Mar refers patients with abnormal FIT results to one of several community GI practices. In 2018, the Sea Mar quality improvement manager requested records from the GI practices of all patients who had had an abnormal FIT result and a GI referral, but no evidence of a completed colonoscopy; these records were in the EHR and included in our analyses. Despite these efforts, we cannot be sure that all records of completed colonoscopies were in the EHR.

### Study procedures

The Sea Mar EHR analyst identified all patients aged 50–75 years with an abnormal FIT result between August 5, 2017 and August 4, 2018 (N = 711). Chart abstraction was performed after August 4, 2018, allowing at least one year of follow-up time between the abnormal FIT result and chart abstraction. No exclusions were applied.

A study team member (AFP) created chart abstraction forms in REDCap (Nashville, TN), based on forms used in previous research [5]. The forms were reviewed by the Principal Investigator (GDC) and pilot-tested by the study analyst and a chart abstractor (MS, AKS). Chart abstraction was carried out by two abstractors, a research team member and a clinic staff member. All chart abstraction was completed on-site at a Sea Mar clinic, using the EHR (Allscripts, Chicago, Illinois). Chart abstractors reviewed scanned documents, provider orders, free text fields, and any other data not captured using automated queries. Sensitivity analyses were not completed as we assumed that missing data were truly missing and treated the chart review as the reference standard.

### Study outcomes

Outcomes included rates of referral to colonoscopy, completion of a pre-procedure visit, completion of a follow-up colonoscopy procedure, and time to follow-up colonoscopy completion. Pre-procedure visit reports were present in 242 of the 711 charts. Because we had no way of knowing whether a pre-procedure visit had been recommended by the GI practice, for the purposes of statistical analysis, we counted all patients with a completed colonoscopy as having completed a pre-procedure visit. This resulted in an additional 123 patients classified as having a pre-procedure visit. We report associations between patient demographic and clinical characteristics and completion of the pre-procedure visit and the colonoscopy. When health record documentation provided reasons that steps were not completed, we recorded that information for analysis.

### Statistical analysis

We report frequencies of socio-demographic characteristics of our study sample. Among the 642 patients who received a referral, we calculated the proportions who completed (1) a pre-procedure visit and (2) a follow-up colonoscopy overall and by referring GI practice. For the 309 patients who completed a colonoscopy, we also calculated median time and interquartile range for colonoscopy completion overall and by GI practice. We used multivariate logistic regression to calculate the association between patient characteristics and completion of a pre-procedure visit and of colonoscopy. Patient characteristics included age, sex, race, ethnicity, preferred language, marital status, insurance type, and number of clinic visits in the year prior to the abnormal FIT. We report adjusted odds ratios and 95% confidence intervals. We also report the concordance between EHR codes and chart abstraction in rates of referral and colonoscopy completion using EHR codes, and rates obtained during chart abstraction. All analyses were performed using SAS version 9.4 (Cary, NC).

## Results

A total of 711 patients had an abnormal FIT result during the 365-day catchment interval and were included in analyses (Table 1). Patients had a mean age of 61 and were predominantly non-Hispanic white (83%); 81% preferred speaking English. Ninety-one percent had health insurance, and 72% had at least one clinic visit in the year prior to their abnormal FIT result.

**Table 1:** Characteristics of patients with abnormal FIT result (N = 711)

Characteristic	Total sample (N = 711)		Completed pre-procedure visit (N = 365)			Completed colonoscopy (N = 309)		
	N	%	N	%^a^	OR (95% CI)	N	%	OR (95% CI)
Age (mean, SD)	61.3 (6.4)							
50–64	486	68.4	262	53.9	Ref	224	46.1	Ref
65–75	225	31.7	103	45.8	**.43 (.25-.72)**	85	37.8	**.46 (.27–.79)**
Sex								
Male	391	55.0	197	50.4	Ref	169	43.2	Ref
Female	320	45.0	168	52.5	1.09 (.79–1.50)	140	43.8	1.05 (.76–1.44)
Race								
White	529	74.4	269	50.9	Ref	229	43.3	Ref
Asian	35	4.9	17	48.6	.93 (.41–2.10)	13	37.1	.71 (.31–1.63)
Black/African American	31	4.4	18	58.1	1.77 (.81–3.86)	17	54.8	1.97 (.91–4.24)
Other/more than 1 race	90	12.7	48	53.3	.86 (.51–1.46)	38	42.22	.75 (.44–1.29)
Not reported	26	3.7	13	50.0	–	12	46.2	–
Ethnicity^b^								
Hispanic/Latino	121	17.0	74	61.2	1.56 (.79–3.10)	62	51.2	1.26 (.64–2.47)
Non-Hispanic/Latino	588	82.7	290	49.3	Ref	246	41.8	Ref
Language								
English	575	80.9	281	48.9	Ref	238	41.4	Ref
Non-English	136	19.1	84	61.8	1.67 (.90–3.13)	71	52.2	1.71 (.92–3.18)
Marital status								
Single	356	50.1	179	50.3	Ref	153	43.0	Ref
Married	222	31.2	114	51.4	1.03 (.70–1.51)	97	43.7	1.03 (.71–1.51)
Separated/divorced/widowed	133	18.7	72	54.1	1.26 (.82–1.92)	59	44.4	1.15 (.75–1.76)
Insurance								
Medicare	135	19.0	72	53.3	**4.19 (1.83–9.58)**	59	43.7	**2.96 (1.29–6.80)**
Commercial	131	18.4	67	51.2	**2.22 (1.08–4.57)**	58	44.3	1.91 (.93–3.93)
Medicaid	380	53.5	198	52.1	1.96 (.98–3.92)	167	44.0	1.57 (.79–3.15)
Uninsured	65	9.1	28	43.1	Ref	25	38.5	Ref
Clinic visits in year prior to Index FIT result^c^								
0	203	28.6	77	37.9	Ref	62	30.5	Ref
1	100	14.1	52	52.0	**1.72 (1.04–2.86)**	44	44.0	**1.73 (1.03–2.90)**
2–3	185	26.0	98	53.0	**1.82 (1.19–2.78)**	87	47.0	**1.94 (1.29–3.08)**
4+	223	31.4	138	61.9	**2.53 (1.67–3.82)**	116	52.0	**2.38 (1.56–3.62)**

Bolded text indicates statistical significance

^a^All patients with completed colonoscopy were assumed to have completed a pre-procedure visit

^b^2 patients did not report ethnicity, percentages may not total 100%

^c^Mean clinic visits in past year = 2.72 (standard deviation = 2.91)

Compared to patients aged 50–64 years, patients aged 65 years and older had lower odds of completing a pre-procedure visit (OR: 0.43; 95% CI 0.25, 0.72) and of completing a colonoscopy (OR: 0.46; 95% CI: 0.27, 0.79). Insurance status was associated with pre-procedure visit completion: patients with commercial insurance or Medicare had significantly higher odds of pre-procedure visit completion than patients who were uninsured (commercial insurance OR: 2.22; 95% CI: 1.08, 4.57; Medicare OR: 4.19; 95% CI: 1.83, 9.58); rates were also higher for Medicaid-enrolled patients, but the difference did not reach significance (OR: 1.96; 95% CI: 0.98, 3.92). Medicare-enrolled patients also had a higher-odds of completing a colonoscopy than uninsured patients (OR: 2.96; 95% CI: 1.29, 6.80). Having a clinic visit in the year prior to the abnormal FIT result was associated with higher odds of pre-procedure and colonoscopy completion, with the strength of these associations increasing with increasing numbers of visits. Neither sex, race, ethnicity, language, or marital status were significantly associated with completion of a pre-procedure visit or a colonoscopy procedure.

When we compared the concordance of data obtained from automated EHR queries and chart abstraction, we observed high concordance between the two data sources on colonoscopy referral (92%) and colonoscopy completion (96%). Automated EHR query data showed that of the 711 patients with an abnormal FIT, 83% were referred to GI, and 42% completed a colonoscopy. In chart-abstracted data, we found evidence that 90% were referred to GI and 43% completed a colonoscopy.

**Fig. 1:**
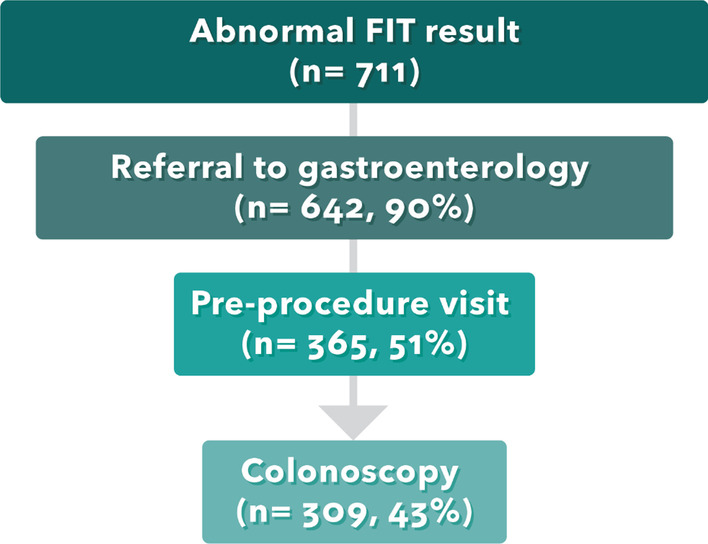
Number and proportion of patients that received each step in the colonoscopy completion process within 12 months of an abnormal fecal immunochemical test (FIT) result. Of 711 patients who received an abnormal FIT result, 90% were referred to gastroenterology, 51% attended a pre-procedure visit with a gastroenterologist, and 43% completed a colonoscopy within 12 months.

**Fig. 2:**
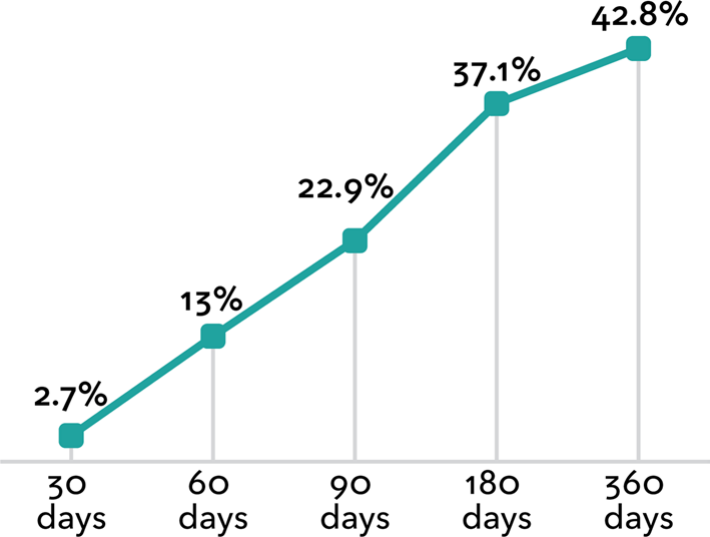
Time from abnormal fecal immunochemical test (FIT) result to colonoscopy receipt, among 711 patients with an abnormal FIT result. Among patients with an abnormal FIT result, 22.9% completed a colonoscopy within 90 days, 37.1% completed a colonoscopy within 180 days, and 42.8% completed a colonoscopy within 360 days

While 90% of patients with abnormal FIT were referred to a GI clinic, only 52% attended a pre-procedure visit and 43% completed a colonoscopy within the year following their abnormal FIT result (Fig. 1). Rates of follow-up colonoscopy completion ranged from 41 to 55% across the 10 most frequently referred GI practices (Table 2). The proportion of patients who completed a colonoscopy over time is shown in Fig. 2. Among the 309 patients who completed a colonoscopy, median time to completion was 83.0 days, ranging from 75.0 days to 112.5 days across the 10 GI practices.

**Table 2:** Colonoscopy completion by gastroenterology facility (n = 642 referred adults)

Gastroenterology facility^a^	Referred N	Completed pre-procedure visit N (%)^b^		Colonoscopy completed N (%)^b^		Time to colonoscopy^c^ Median days (IQR)	
Overall	642	365	(56.9)	305	(43.5)	83.0	(52–131)
Facility 1	94	52	(55.3)	49	(52.1)	90.0	(44–125)
Facility 2	31	21	(67.7)	16	(51.6)	75.0	(49.5–119.5)
Facility 3	56	26	(46.4)	24	(42.9)	84.5	(57.5–162)
Facility 4	53	34	(64.2)	25	(47.2)	90.0	(68–133)
Facility 5	26	13	(50.0)	12	(46.2)	87.0	(68–129.5)
Facility 6	57	34	(59.7)	27	(47.4)	79.0	(39–122)
Facility 7	36	21	(58.3)	16	(44.4)	76.5	(64.5–155.5)
Facility 8	40	27	(67.5)	22	(55.0)	112.5	(73–144)
Facility 9	73	31	(42.5)	30	(41.1)	85.5	(61–133)
Facility 10	69	44	(63.8)	37	(53.6)	86.0	(51–115)
Other/Unknown	96	62	(57.9)	51	(47.7)	70.0	(45–138)

^a^Initial referral location, patients may have transferred care between referral and pre-procedure visit/colonoscopy. Unknown facilities have been excluded from this table; percentages may not total 100

^b^Percentages are based on the number referred for a given practice

^c^Median time among those who completed a colonoscopy

Among the 711 patients who had an abnormal FIT, 402 (57%) did not complete a colonoscopy; 69 (10% of the 711) received no referral, 277 (39%) received a referral but did not complete a pre-procedure visit, and 56 (8%) completed a pre-procedure visit but did not complete a colonoscopy. A reason for not completing the colonoscopy was present in 246 charts (61% of those who did not complete a colonoscopy); reasons provided are summarized in Table 3. The most common reasons were that the patient declined, missed, or canceled an appointment (19%) or the provider was unable to reach the patient (15%).

**Table 3:** Summary of chart-abstracted reasons for not completing a given step in the screening pathway

Total N with a reason^a^	No referral n = 69		No pre-procedure visit n = 277		No colonoscopy n = 56		Overall N = 402	
Reasons	N	%	N	%	N	%	N	%
Patient declined, no-show, canceled	**13**	**18.8**	**57**	**20.6**	**7**	**12.5**	77	19.2
Provider unable to reach patient	4	5.8	**53**	**19.1**	2	3.6	59	14.7
Competing health concerns/ end-of-life/ deceased	**11**	**15.9**	**15**	**5.4**	5	8.9	31	7.7
Patient barriers (e.g. transportation, insurance, unstable housing)	3	4.3	13	4.7	3	5.4	19	4.7
Patient given a 2nd FIT	**8**	**11.6**	10	3.6	0	0.0	18	4.5
Recent colonoscopy	6	8.7	2	0.7	**6**	**10.7**	14	3.5
GI authorization/ clearance not obtained, inadequate prep^b^	2	2.9	5	1.8	**7**	**12.5**	14	3.5
Patient moved/ transferred care	2	2.9	7	2.5	0	0.0	9	2.2
Unknown—no indication in chart	**21**	**30.4**	**111**	**40.1**	**24**	**42.9**	**156**	**38.8**

Bolded text indicates top four reasons for each step

^a^A given patient’s chart could have more than one reason

^b^Includes 1 chart noting that the PCP did not follow-up with patient

## Discussion

Using medical record abstraction, we assessed patterns of follow-up colonoscopy completion among patients of a large, diverse FQHC in Western Washington who received an abnormal FIT result. While 90% of these patients were referred to colonoscopy, only 52% attended a pre-procedure visit and 43% completed a colonoscopy within the year following their abnormal FIT result, well below the national benchmark of 80% [8]. Attrition was highest in the intermediate step between the GI referral and the pre-procedure visit (38% percentage point drop). Efforts to improve follow-up colonoscopy rates in FQHCs might focus on supporting the care transition from primary care to specialty gastroenterology care. This may be particularly needed for patients aged 65–75, those without a clinic visit in the prior year, and those who lack health insurance.

Given that adults with an abnormal FIT result have a 4–9% chance of having undiagnosed colorectal cancer and a 2.4-fold elevated risk of dying from the disease than those with normal FIT results [8, 16–19], efforts are needed to improve follow-up colonoscopy rates in these settings. Prior studies have documented six-month FQHC follow-up colonoscopy completion rates between 18 and 57%, and our rate of 37% was within that range [3, 4, 20]. Our observed 43% one-year colonoscopy completion rate was lower than that found in three prior studies in similar settings (53–58%): our team previously reported a 53% one-year follow-up colonoscopy rate in a sample of 2,018 patients from eight non-integrated FQHCs [21], and two other studies, Issaka and colleagues’ study in San Francisco and one in Dallas, found one-year completion rates of 56% and 58%, respectively [6, 20]. However, patients in the San Francisco and Dallas studies were served by integrated safety-net systems that generally referred to a single network hospital, which likely facilitated both colonoscopy completion and data capture [6, 20].

The median time to follow-up colonoscopy that we observed, 83 days, was within the range reported in prior studies (64 days to 184 days) [4–6, 20]. Our observed time to colonoscopy was within the 90-day benchmark set by the PROSPR initiative [22], suggesting that patients who successfully access GI services generally are able to obtain a timely procedure. Notably, we observed a 37.5-day variation in median time to follow-up colonoscopy across GI referral sites, (range = 75 to 112.5 days).

Nearly 40% of patients with no follow-up colonoscopy had no reason documented in their medical record. Among those for whom a reason was documented, the most common reasons were that the patient declined, missed, or canceled an appointment, or could not be reached. These findings were similar to those of prior research by our team showing that common reasons were a patient’s declination or appointment no-show (55%), having a recent prior colonoscopy (22%), or that the patient could not be reached (13%) [21]. Our current study extends these findings by specifying the distribution of reasons for each intermediate step. Specifically, our findings highlight the importance of competing health concerns and being given a second FIT (to repeat the test) as key barriers for patients who did not receive a referral; and of having had a recent colonoscopy, lacking insurance authorization/ medical clearance, or having inadequate bowel prep as key barriers for patients with who completed a pre-procedure visit but did not receive a colonoscopy. Notably, these reasons are not always patient-reported, and leave unanswered questions about why patients may have declined or failed to show up for an appointment. Such information could be obtained from direct patient interviews. Moreover, the low proportion of charts with documented reasons for no colonoscopy may underscore the need to promote informed discussions between providers and patients about the risk of late cancer detection among patients who discontinue the screening process [6]. Improving the documentation of reasons for no follow-up could better inform future interventions to improve follow-up care.

The substantial drop-off that occurred between the referral and the pre-procedure visit raises the question of whether the pre-procedure visit creates unnecessary burden for patients to attend colonoscopy procedures. Further research is needed to address this question. Given the recent and rapid adoption of telehealth as a replacement for in-person visits during the COVID-19 pandemic, this question could potentially be answered using a natural experiment comparing colonoscopy completion rates among patients attending telehealth pre-procedure visits and in-person pre-procedure visits.

Our findings have important implications for the design and adaptation of patient navigation programs and other interventions to address barriers to completing follow-up colonoscopy. First, our findings suggest that efforts are most needed to support referred patients in establishing care with GI practices. These supports are particularly needed for patients aged 65 and older, those who lack health insurance, and those who have not attended a clinic visit in the year prior to an abnormal FIT result. Prediction modeling could be useful to further identify patients who may benefit most from such supports [15]. Moreover, our findings show that while fewer than half of patients with an abnormal FIT completed a colonoscopy, time to colonoscopy completion overall was within the timeframe of national benchmarks, possibly suggesting an absence of prolonged scheduling delays among those who successfully access care.

Innovations in non-invasive screening technology, such as urine tests, exhaled breath tests, and blood-based tests, could minimize patient stress and discomfort and improve adherence to CRC screening [23, 24]. However, research is scarce on the performance of these innovations to identify patients with advanced adenomas [24]. Irrespective of first-line test choice, efforts will likely still be needed to assure that those with limited access to health care can get a follow-up colonoscopy.

### Strengths and limitations

A primary strength of this study is our large retrospective sample from a non-integrated FQHC that refers patients to several community GI practices. Additionally, our research team conducted complete health record abstraction, and we report findings across multiple steps in the colonoscopy process. Nevertheless, our study has several limitations. While our study relied on chart-abstracted data and requests of colonoscopy records from GI practices, we cannot be sure that all records of completed colonoscopies were in the EHR; thus, our reported colonoscopy completion rates may underestimate true rates. However, given the high concordance between EHR-query-obtained and chart-abstracted results (92% for GI referral; 96% for colonoscopy completion), we can be reasonably confident our data include nearly all procedures. Our analysis was also limited to information in the health record, which inherently omits important system-level factors, such as referral tracking procedures, protocols to follow up with patients, health record interoperability, and available staffing resources [25]. Moreover, reasons for non-completion recorded in the EHR are incomplete and we did not gather qualitative data directly from patients who did not complete the recommended colonoscopy. Finally, our findings are from a single FQHC in Western Washington and may not generalize to other settings.

## Conclusions

Despite the elevated risk of CRC among individuals with abnormal FIT results, rates of follow-up colonoscopy in a non-integrated FQHC were substantially below the 80% target set by the Multi-Society Task Force on Colorectal Cancer. Efforts to improve rates of follow-up colonoscopy might focus on supporting the care transition between primary to specialty GI care, and emphasizing care for patients aged 65–75 years, those without a recent clinic visit, and those who lack health insurance.

## Data Availability

The datasets analyzed during the current study and the full trial protocol are available from the corresponding author on reasonable request.

## Abbreviations

CRC: Colorectal cancer
FIT: Fecal immunochemical test
FQHC: Federally qualified health center
EHR: Electronic health record
GI: Gastroenterology
